# Expanding the evidence for cross-sector collaboration in implementation science: creating a collaborative, cross-sector, interagency, multidisciplinary team to serve patients experiencing homelessness and medical complexity at hospital discharge

**DOI:** 10.3389/frhs.2023.1124054

**Published:** 2023-09-08

**Authors:** Amanda Joy Anderson, Katia Noyes, Sharon Hewner

**Affiliations:** ^1^ School of Nursing, State University of New York at Buffalo, Buffalo, NY, United States; ^2^ Division of Health Services Policy and Practice, Department of Epidemiology and Environmental Health, State University of New York at Buffalo, Buffalo, NY, United States

**Keywords:** cross-sector collaboration, intersectoral collaboration, medical respite care, implementation science, people experiencing homelessness, care coordination, high-need

## Abstract

**Introduction:**

Patients with medical and social complexity require care administered through cross-sector collaboration (CSC). Due to organizational complexity, biomedical emphasis, and exacerbated needs of patient populations, interventions requiring CSC prove challenging to implement and study. This report discusses challenges and provides strategies for implementation of CSC through a collaborative, cross-sector, interagency, multidisciplinary team model.

**Methods:**

A collaborative, cross-sector, interagency, multidisciplinary team was formed called the Buffalo City Mission Recuperative Care Collaborative (RCU Collaborative), in Buffalo, NY, to provide care transition support for people experiencing homelessness at acute care hospital discharge through a medical respite program. Utilizing the Expert Recommendations for Implementing Change (ERIC) framework and feedback from cross-sector collaborative team, implementation strategies were drawn from three validated ERIC implementation strategy clusters: 1) Develop stakeholder relationships; 2) Use evaluative and iterative strategies; 3) Change infrastructure.

**Results:**

Stakeholders identified the following factors as the main barriers: organizational culture clash, disparate visions, and workforce challenges related to COVID-19. Identified facilitators were clear group composition, clinical academic partnerships, and strategic linkages to acute care hospitals.

**Discussion:**

A CSC interagency multidisciplinary team can facilitate complex care delivery for high-risk populations, such as medical respite care. Implementation planning is critically important when crossing agency boundaries for new multidisciplinary program development. Insights from this project can help to identify and minimize barriers and optimize utilization of facilitators, such as academic partners. Future research will address external organizational influences and emphasize CSC as central to interventions, not simply a domain to consider during implementation.

## Introduction

Cross-sector collaboration (CSC) refers to the complex process of providing services through a collaborative framework of multiple agencies that a single agency could not achieve alone (1). Despite extensive use in organizational research and compelling demand to meet the care delivery for patients with medical and social complexity, CSC is a strategy that only recently began to emerge in health services implementation research. Previous studies documented that CSC has been employed in efforts to improve care transitions for people with serious mental illness (2), prevent infectious diseases (3), address obesity and non-communicable diseases (4), and advance health-promoting policy (5). Because of organizational complexity, differences in goals and financial models across agencies (6) and exacerbated social and clinical needs of patient populations receiving care requiring CSC (7), implementation and sustainability of CSC interventions remains poorly understood. Furthermore, the reliance on the traditional, disease- or illness-based biomedical care model in most of the US healthcare settings (8) often results in medical agencies leading CSC efforts, quality improvement, and innovation, which may compromise integration across agencies, reduce effectiveness, and hinder long-term sustainability of cross-sector interventions (9).

An example of a population with needs that demand collaborative care from cross-sector, interagency, multidisciplinary teams, is people experiencing homelessness at acute care hospital discharge. Compelling evidence from the last decade of health services research has demonstrated increasing medical and social complexity of people experiencing homelessness (10–13). In addition, the recent push toward community-based medical management means that patients are discharged from hospitals sooner, and with more complex treatment needs that they must manage at homes that they do not have (14). In parallel to patients' growing medical needs, our understanding of the impact that social factors play on their overall wellbeing and experience of care is also growing (15). We now know that social determinants of health play a larger part than we have historically accounted for in how and when patients access care, their trust in clinicians, whether they have the capacity to follow treatment plans, and if they will successfully transition to the community after acute hospitalization events (16). With high rates of housing insecurity, financial strains directly linked to healthcare cost, and demands of personal relationships and responsibilities, social factors often lead to patients' premature return to hospital. Studies show that lack of support at home, income limitations, and transportation demands are often more impactful in causing patients to decide to return to the hospital than clinical symptoms—realities that are exponentially worse in people experiencing homelessness at hospital discharge (17).

While several studies used the CSC approach to address care needs of complex patients, only a few demonstrated positive results (18). A prominent example is the study out of Camden, NJ which delivered community-based care coordination to high-need patients with patterns of high health services utilization (19). The study intervention, while rigorous in its attempt to address health and social needs, was primarily delivered from a single organizational entity, and lack of significant impact on rehospitalizations confirms the need for targeted CSC for high-need populations. In contrast, a growing body of health and social science literature from the National Institute of Medical Respite Care (20), a subsidiary of the Healthcare for the Homeless Council, attests to the multi-faceted and successful approach to care transition delivery, known as medical respite care. In the United States, medical respite programs provide support to individuals experiencing homelessness and medical complexity at the time of hospital discharge and have the capacity to facilitate linkages between health and social sector organizations (21, 22). However, little is known about implementation of medical respite programs, and the evidence of successful implementation is scarce.

The lack of insight into CSC in general, and as a specific strategy to facilitate care transitions for people experiencing homelessness, poses implementation challenges for programs reliant upon collaborative service delivery. The aim of this study was to outline barriers and facilitators to the implementation and sustainability of a program based on a team of cross-sector providers. The social services-based program serves people experiencing homelessness in Buffalo, NY.

## Methods

### Setting

The Buffalo City Mission (BCM) is Buffalo's largest homeless shelter, with capacity to serve 200 men, women, and children in their emergency and transitional shelters at two downtown locations, the Alfiero Family Center for men, and the Cornerstone Manor, for women and children. At the time of initiation of this collaborative project, the BCM was transitioning from an existing facility to a larger men's facility that included a 13-bed unit for medical respite care, to be called the Recuperative Care Unit (RCU).

The BCM receives referrals to its RCU program from regional acute care hospitals. Most patients come from the county acute care hospital. The hospital also maintained an existing contractual post-acute program for behavioral health patients requiring BCM services and county crisis services oversight. BCM staff only provides social services to the tenants and has formed partnerships with other collocated agencies to provide other necessary services: a Federally Qualified Healthcare Center (FQHC) primary care agency, and a behavioral health agency. Additional collaborative partners include specialty healthcare providers, transportation providers, and legal agencies as dictated by individual patient needs and located elsewhere in the city.

### Participant sample

**Figure 1:**
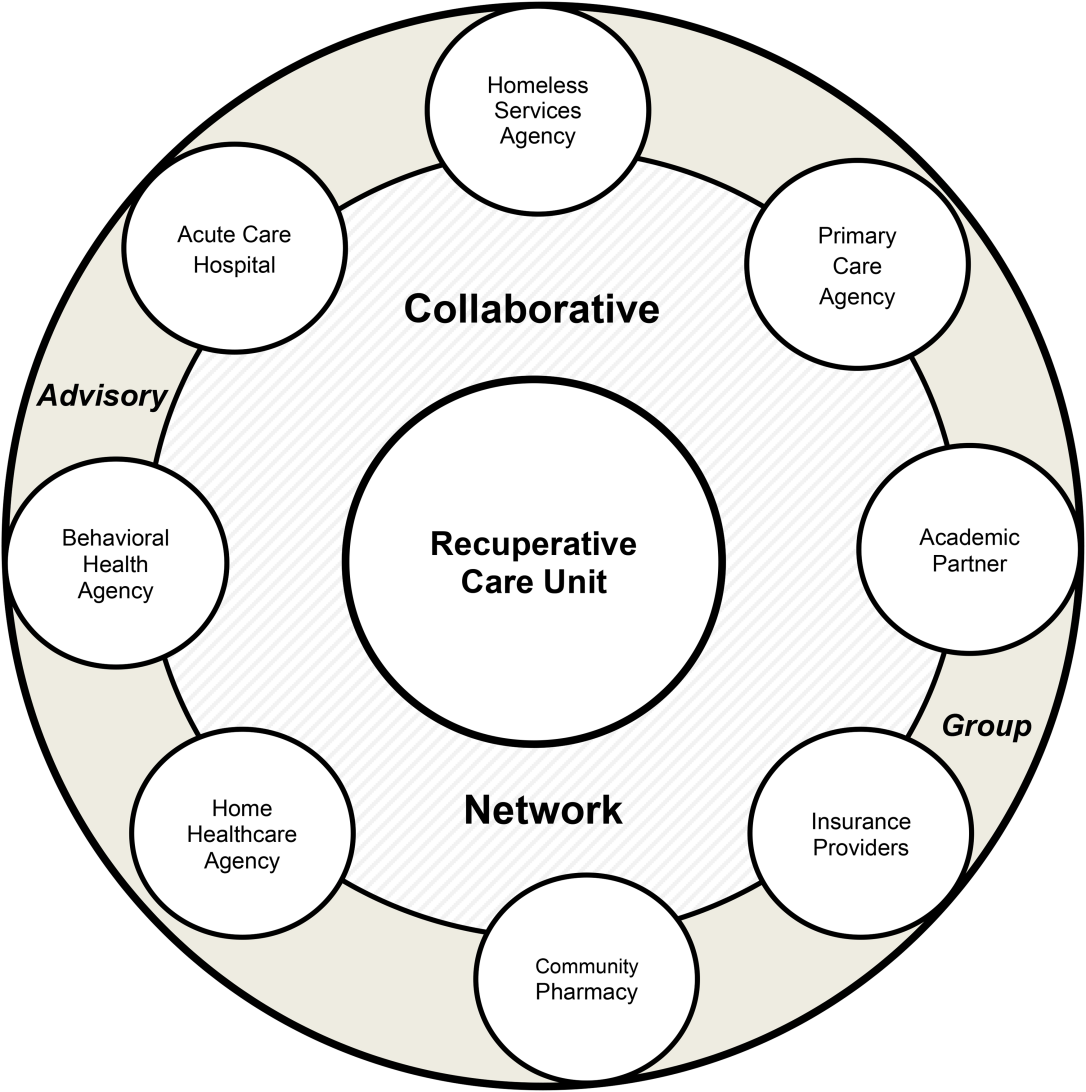
Expanded RCU collaborative model.

The study participants include project representatives from social, behavioral, and academic agency partners represented in Figure 1 (*N* = 10–15 primary agencies), with individuals from a mixture of frontline, provider, academic, and administrative departments, and roles.

Through the support of a grant procured from the Robert Wood Johnson Foundation Clinical Scholars Fellowship (RWJF 77883), a partnership between the Buffalo City Mission and the State University of New York at Buffalo School of Nursing (UBSON) was formed, followed by a collaborative, cross-sector, interagency, multidisciplinary team of partners making up the study sample (Figure 1) known as the Buffalo City Mission Recuperative Care Collaborative medical respite program (RCU Collaborative). The fellowship, which includes leadership enrichment and project management training (23), launched in September 2021, and scholars joined with organizational stakeholders to form the RCU Collaborative for the purpose of opening the medical respite program within a cross-sector framework.

### Study design

To facilitate the creation of the collaborative, cross-sector, interagency, multidisciplinary team, the project stakeholders agreed upon a schedule of recurring meetings including a weekly case conference of frontline providers, a monthly advisory group of administrators, and *ad hoc* workgroups for operational and programmatic development needs (Table 1). Members from each partnered organization attended recurring meetings and were called upon for workgroup-specific tasks.

**Table 1:** RCU collaborative meeting structure.

Meeting	Cadence	Description
Clinical Scholar	Weekly	Fellow-only think-tank meeting for reflection, cross-communication, and brainstorming
Advisory Group	Monthly	Cross-sector leadership stakeholder meeting for review and approval of workgroup and case conference output; new strategy and alignment
Operations Workgroup	Weekly	Frontline cross-sector group charged with policy, procedure creation
Case Conference	Weekly	Cross-sector clinical review of respite clients for purpose of care transition management through program
Learning Consortium	Monthly	National Health Care for Homeless Council Medical Respite Learning Consortium for new medical respite providers
CS Retreats	Quarterly	Quarterly RWJF Clinical Scholar leadership retreats to support professional development of fellows

### Data analysis

The study data were generated through informational interviews, review of regular meeting materials and operational procedures, and feedback from involved team members. The study team analyzed study data, identified reported barriers to implementation of collaborative, cross-sector, interagency, multidisciplinary team, and mapped them to appropriate implementation strategies using the Expert Recommendations for Implementing Change (ERIC), a validated framework of implementation strategies (Table 2) (24, 25). The preferences were given to implementation strategies that were aligned with implementation facilitators identified by the study informants.

**Table 2:** ERIC Implementation strategies by cluster with project-specific emphasis.

Implementation strategy cluster name	Implementation strategies
Use and evaluate iterative strategies	Asses for readiness and identify barriers and facilitators; Audit and provide feedback; Purposefully reexamine the implementation; Develop and implement tools for quality monitoring; Develop and organize quality monitoring systems; Develop a formal implementation blueprint; Conduct a local need assessment; Stage implementation scale up; Obtain and use patients/consumers and family feedback; Conduct cyclical small tests of change
Provide interactive assistance	Facilitation; Provide local technical assistance; Provide clinical supervision; Centralize technical assistance; Provide clinical supervision; Centralize technical assistance
Adapt and tailor to context	Tailor strategies; Promote adaptability; Use data experts; Use data warehousing techniques
Develop stakeholder interrelationships	Identify and prepare champions; Organize clinician implementation team meetings; Recruit, designate, and train for leadership; Inform local opinion leaders; Build a coalition; Obtain formal commitments; Identify early adopters; Conduct local consensus discussions; Capture and share local knowledge; Use advisory boards and workgroups; Use an implementation advisor; Model and simulate change; Visit other sites; Involve executive boards; Develop an implementation glossary; Develop academic partnerships; Promote network weaving
Train and educate stakeholders	Conduct ongoing training; Provide ongoing consultation; Develop educational materials; Make training dynamic; Distribute educational materials; Use train the trainer strategies; Conduct educational meetings; Conduct educational outreach visits; Create a learning collaborative; Shadow other experts; Work with educational institutions
Support clinicians	Facilitate relay of clinical data to providers; Remind clinicians; Develop resource sharing agreements; Revise professional roles; Create new clinical teams
Engage consumers	Involve parents/consumers and family members; Intervene with patients/consumers to enhance uptake and adherence; Prepare patients/consumers to be active participants; Increase demand; Use mass media
Utilize financial strategies	Fund and contract for the clinical innovation; Access new funding; Place innovation on fee for service lists/formularies; Alter incentive/allowance structures; Make billing easier; Alter patient/consumer fees; Use other payment schemes; Develop disincentives; Used capitated payments
Change infrastructure	Mandate change; Change record systems; Change physical structure and equipment; Create or change credentialing and/or licensure standards; Change service sites; Change accreditation or membership requirements; Start a dissemination organization; Change liability laws

Adapted and cited from (25).

## Results

The primary goal of this study was the creation of a new collaborative, cross-sector, interagency, multidisciplinary team delivering medical respite care to people experiencing homelessness after acute care hospital discharge, the RCU Collaborative. Below we describe barriers and facilitators to implementation of RCU Collaborative model of care (outlined with detail in Table 3) and propose implementation strategies to overcome these barriers by maximizing strengths and resources identified by the members of the Collaborative.

**Table 3:** RCU collaborative partners and care actions by transition phase.

Organization (Facilitator)	Phase 1		Phase 2	Phase 3
	Hospital discharge	Respite admission	Respite days 1–30	Respite discharge
Acute Care Hospital (Discharge Planners)	Completes online referral form/sends clinical documentation; Schedules new patient visit at respite PCP; Refers patient to collaborative HHA/BHA	Troubleshoots post-discharge transition needs with HSA, PCP, AP	Participates in Weekly Case Conference	Confirms discharge and conveys to internal billing management
			Troubleshoots care needs with HSA, PCP, AP as applicable to hospitalization or prevention of readmission	
Homeless Service Agency (Respite Case Managers)	Evaluates referral elements from ACP; Requests PCP; AP referral review; Requests ACH clarification/documents/visit; Denies/accepts patient	Confirms PCP, HHA, BHA linkages, discharge elements	Provides 24–7 oversight/assistance to admitted patients; Generates and reviews daily census, individual patient plans; Facilitates necessary care escalation, coordination, disposition changes; Leads Weekly Case Conference; Leads Monthly Advisory Group	Transition patient to disposition decided upon by collaborative decision; Communicate with PCP, referred agencies for transition of care; Communicate with ACH to close billing for patient
		Begins wraparound case management protocol		
Primary Care Agency (Physician's Assistant)	Reviews clinical elements of referral documents	Confirms new patient linkage and first visit with HSA	Facilitates post-discharge patient follow up within 7 days; Manages medical escalations 24–7 as necessary; Participates in Case Conference Group; Sends administrative representative to Monthly Advisory Group	Confirms disposition location and plan for care after transition
Home Health Agency (Home Health Providers)		Confirms new patient linkage and first visit with HSA	Facilitates nursing, PT/OT services; Participates in Case Conference Group; Sends administrative representative to Monthly Advisory Group	Confirms disposition location and plan for care after transition
Behavioral Health Agency (Behavioral Health Providers)		Confirms new patient linkage and first visit with HSA	Facilitates behavioral health/substance abuse services; Participates in Case Conference Group; Sends administrative representative to Monthly Advisory Group	Confirms disposition location and plan for care after transition
Academic Partners (Clinical Scholars)	Assists with patient referral review		Assists with cross-sector connections, troubleshooting; Assists with facilitation of Weekly Case Conference; Sends administrative representative to Monthly Advisory Group	Assists with patient data tracking
Case Conference Group (Frontline Facilitators)	Discusses new patient referrals	Troubleshoots post-discharge transition needs	Meet within first 7 days of patient admission (weekly recurrence); Address discharge gaps as necessary, health and social care needs; Assess rehospitalization risk/need for level care	Collaboratively decide upon patient discharges and transfers out of program
Advisory Group (Administrative Representatives)			Addresses policy & practice needs; Facilitates accountability across organizations; Serves as feedback and approval mechanism for frontline providers	

ACH, Acute Care Hospital; HSA, Homeless Service Agency; PCP, Primary Care Agency; HHA, Home Health Agency; BHA, Behavioral Health Agency; AP, Academic Partners

### Implementation barriers

#### Organizational culture clash, disparate visions & workforce challenges

Unclear communication between primary partner leadership, complicated by historically strained relationships between partnering organizations, and unclear roles at the start of our initiative resulted in culture clash between organizational leaders. Additional barriers stemmed from differences in policy and procedure between organizations, which were tied to organizational culture, size, values, and understanding of healthcare service delivery. The lead homeless service agency functioned within a faith-based framework, with administrative restrictions on funding mechanisms that limited its operating strategies. Additionally, the reliance on relational workarounds and top-down administrative hierarchy for decision making in the lead social sector agency, caused barriers to formal operating procedure implementation with healthcare entities accustomed to more protocol-driven operating mechanisms (26). Extensive workforce turnover and leadership changes in the leading social sector agency led to persistent barriers to implementation and program growth. Additionally, the slow utilization of the respite program by regional organizations can be attributed to the impact of the COVID-19 pandemic, as regional acute care hospitals and health departments facilitated care transitions through external mechanisms, and shelter policy prohibited admittance of new patients who were actively infected with the virus.

#### Related ERIC strategy cluster: use and evaluate iterative strategies

An early element of the RCU Collaborative implementation aimed at restoring trust in relationships came with the creation of relational meeting structure, which fosters frequent and high-quality communication, facets of relational coordination inherent to successful cross-sector partnerships (1, 27), and an evidence-based strategy in care coordination programs for high-risk patients (28). The ERIC elements of *assess for readiness and identify barriers and facilitators* was done through an administrative-frontline dyad, the RCU Collaborative launched two key meetings to facilitate restored trust in relationships through consistent forums: the RCU Weekly Case Conference and the RCU Advisory Group (Table 1).

Another strategy from this cluster included *development of a formal implementation blueprint*, which included structures such as the RCU Weekly Case Conference, where cross-sector team members committed to meet via teleconference to discuss referrals, admissions and current RCU patients, fostered increased communication, discussion about discharge quality, and aligned efforts toward throughput and readmission reduction across the RCU Collaborative. Following each patient for the 30-day period post-discharge, the case conference served as a conduit for relationship building because of the consistent audience across sectors, frequency of communication, and the shared burden of the care transition period with the discharging hospital.

In addition to the case conference, the RCU Collaborative leaders created a project hierarchy and meeting structure that included an approval mechanism forum called the Advisory Group, which was part of the strategy of *developing and organizing quality monitoring systems*. Comprised of partnering organization leaders, the Advisory Group served as a monthly mechanism for strategic decision-making, evaluation of cross-sector concerns, and approval of policies that were being drafted at the frontline level in a separate workgroup and from within the case conference. The dyadic pairing of Advisory Group members with frontline members of the case conference and workgroup provided clear structure for escalation and approval, and allowed frontline members to air concerns with each other, brainstorm solutions, and enact policy upon approval of collective leaders in the Advisory Board and within the relationships sustained by the meeting structure.

#### Related ERIC strategy cluster: change infrastructure

At the launch of the RCU Collaborative, the key element of shared policy structure, or according to ERIC, the infrastructure needed such as *membership requirements, mandate change,* and, *record systems*, that was addressed was the need to set guidelines for RCU patient eligibility criteria, and the process for referring patients to the RCU. This information was crucial to the movement of financial and contractual elements being driven by BCM leadership, and elements were generated within a workgroup comprised of leaders and frontline staff. Additionally, this strategy informed the creation of a robust data collection method that is practical, based on an evidence-based model (29), and rich in information that is often not extractable from health services data. Evidence was drawn from standards set by NIMRC, as well as existing readmission reduction literature, with a focus on Coleman's Care Transitions Intervention criteria (30, 31).

**Figure 2:**
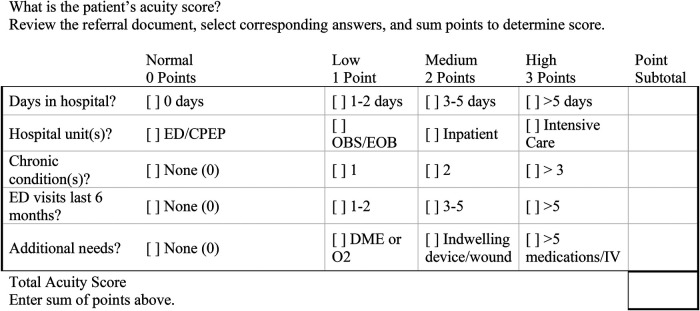
RCU collaborative referral acuity score tool.

The referral process, an element of baseline process change pertinent to *membership requirements* specific to acute care hospital referral expectations, was the first shared policy and procedure element approved by the Advisory Group and represented a process that benefitted from the creation of a support tool, a key element to successful cross-sector collaborations drawn from Accountable Care Organization literature (32). The tool was comprised of a screening and acuity scale based on a published risk index (33), and performed by BCM when patients were referred by acute care discharging providers with unit-placement preference specified (Figure 2). As BCM RCU is located within the compound shelter facility that includes 30-day emergency shelter units and a transitional housing unit, the ability to rank referral acuity for admission to RCU as a shared process was a key policy decision. Once successfully implemented in paper form, the RCU Collaborative designed an online portal for referral, and executed go-live and affiliated training for easier management by both sectors. In tandem with creation of eligibility and referral process and requirements, the RCU Collaborative created a shared Policy & Procedure manual that addressed the elements of cross-sector respite care given to patients during the first seven to thirty days of stay.

Another key aspect of the shared policy and procedure work, which required cross-sector input, was the specified escalation procedures for discharge, clinical, behavioral, and mental health emergencies, which could fall into the ERIC strategy in this cluster, *mandate change*. Since the RCU was housed within BCM and did not offer or employ onsite clinical service providers, the RCU Collaborative created a collaborative algorithm with program partners to manage urgent needs. With the collective goal of avoiding rehospitalization or emergency room utilization, and optimize the nature of non-clinical RCU staff, the escalation policy provided step-by-step instruction in a visual format. For example, if a patient was admitted without durable medical equipment listed on discharge instructions, the non-clinical staff would refer to the discharge escalation pathway, which specified how to contact a discharging provider to escalate a discharge need. Likewise, in the event of clinical emergency, the algorithm specified how to utilize the primary care partner, including in off-hours, to resolve non-emergency-level clinical issues that had formerly been deferred to emergency department care by shelter staff.

Finally, the RCU Collaborative established a means for tracking patients during the first year of the program, an example of ERIC strategy *change record systems*. This method was comprised of both automatic and manual extraction from the BCM electronic record known as the Client Record Online Service System (CROSS), a customized product of WellSky. Tracking included patient and programmatic demographics, descriptive metrics to establish population baseline of health and social risk factors, program quality improvement metrics, and the calculation of 7- and 30-day readmission rates.

### Implementation facilitators

#### Clear group composition, academic partnerships & strategic linkages with hospitals

By linking administrative approval with frontline implementation and feedback, we adapted policies to the unique needs of our patients as they arose. This dyadic structure also improved our relational trust and creation of shared policy and procedure, which the collaborative depended on for facilitation of protocol-based, accountable communication and action across agencies. For example, acute care hospital leadership appointed specific middle-management and frontline staff to contribute to the weekly case conference of collaborative providers, which allowed for real-time information exchange on care transition quality and patient needs post-discharge; a rare snapshot that most acute care providers lack access to. This feedback mechanism extended across the entire care continuum from hospital discharge to patient transition out of the medical respite unit, which allowed for role normalization, consistent communication, and access to multiple record systems by network team members to inform a truly holistic dialogue about patient risk and care needs. Four academic-based research team members sponsored by the UBSON-based Robert Wood Johnson Foundation Clinical Scholars project, functioned as Implementation Facilitators in this project, defined by the updated CFIR framework as, “Individuals with subject matter expertise who assist, coach, or support implementation.” (34) These members entered the project in Fall 2020 at grant initiation and included two of the authors (AA; SH). All participants in this group were White females with nursing degrees and greater than ten years of clinical and/or administrative experience.

#### Strategic linkages with acute care hospitals

Through a recurring weekly case conference that included all collaborating partners, and a recurring monthly oversight committee, our model structured a practical, important avenue for communication and feedback that mirrored real-time patient discussions often seen in acute care setting and incorporated representatives from acute care hospitals sending patient referrals as integral stakeholders in the RCU Collaborative. This facet is unique in care transition literature, but crucial to the success of the collaborative, cross-sector, interagency, multidisciplinary team. Our dyadic composition of frontline providers across the care transition continuum, paired with an oversight committee of leaders, was vital to our successes in clarifying cross-sector roles and implementation of the program.

#### Related ERIC strategy cluster: develop stakeholder interrelationships

Clarity of roles across involved organizations is a shared element in administrative theory on cross-sector collaboration, and the ERIC study (1, 24, 25). Although BCM and involved RCU Collaborative stakeholders had historical relationships in care transitions, early work included establishment of a visual model to establish cross-sector roles and connections (Figure 1). The model helped to *build a coalition* to align newly colocated program partners for input on collaborative capacities, and to begin the creation of procedural elements to outline how RCU care would be implemented, and what it would consist of (Table 3). For example, team discussions included licensing limitations for colocated providers in the RCU space, ensuring program expectations such as initial patient care appointments, facilitating communication during urgent situations to reduce rehospitalizations, and resolving discrepancies in discharges. During this time prior to program launch, new partners were added to the model, including stakeholders in community organizations, BCM departments such as dietary, housing coordination, and spiritual care, and home health care organizations. Although the visual model continues to expand and change throughout the course of the project, its value as a grounding tool for clarifying cross-sector organizational and individual roles, was seen early on.

A second strategy cluster element was the use of *academic partnerships* with the UBSON Clinical Scholar partners as boundary-spanning agents within the network. Brokers and boundary-spanners are agents within collaborative networks who work to connect disparate parties for the purpose of collective good (27, 35). The UBSON Clinical Scholars were four nurses working internally to the RCU Collaborative, connected through the RWJF grant elements. One (AA), a PhD student and experienced nurse administrator, performed research assistant duties as an internal member of the frontline BCM team, giving support to case managers and leadership in the creation of the administrative structure and policies. Another (SH), a PhD-prepared care transitions scientist and faculty member, liaised with RCU Collaborative leaders to facilitate the creation of project hierarchy and role clarity. Two additional members worked internally at the contracted acute care hospital, with a proportion of their salaried hours dedicated to the RCU Collaborative project. Their internal knowledge and access to the primary acute provider contributed to boundary-spanning capacities for establishing transitional elements such as discharge and referral criteria expected of hospital discharge planners sending patients to the RCU, and to subsequent clarity of RCU care to hospital stakeholders.

Additional strategy cluster elements include opportunities both utilized and provided by the RCU Collaborative members that *promoted network weaving*. For example, a portion of members of the RCU Collaborative participated in the NHCHC Medical Respite Network Learning Consortium, and Clinical Scholar Fellows learned from leadership activities inherent to the CS program and coaching support. Additionally, BCM team members created materials about the RCU for the acute care hospital partners and rounded in the hospital to teach about eligibility criteria, referral processes, and typical respite stay. Finally, CS Fellows executed multiple “Lunch and Learn” sessions for BCM leadership on topics relevant to RCU such as data for quality improvement training, and financing respite care.

## Discussion

Retrospective reflection of our program launch led to an understanding of the renewed importance of having a clearly defined, shared vision when engaging in a community-based implementation project across several interdisciplinary agencies. While this step is explicitly outlined in many determinant IS frameworks, including the original CFIR framework (34), in practice it is often omitted as unnecessary or too simplistic. Barriers related to how to operate and evaluate the program largely stemmed from varying data use standards among different agencies, differences in quality improvement practices across sectors, and the limitations of social sector record data, which relies heavily on elements required by the Housing and Urban Development documentation and varies in quality due to qualifications of shelter personnel. Additionally, the heavy healthcare influence of the academic partners initially caused barriers in protocol implementation in the social sector, leading to a leadership clash and need to reframe to integrate the healthcare paradigm into the social sector culture, not overtake.

Additional lessons learned include the extreme complexity of measurement and tracking outcomes across a collaborative, cross-sector, interagency, multidisciplinary team of providers using vastly different record keeping systems, some including paper**.** This stems from the lack of insight onto standardized outcomes for medical respite care, and care of people experiencing homelessness and other socially complex presentations. Conventional studies of high-need populations track readmission or utilization reduction, but this practice has since fallen out of favor considering the limitations when addressing patients experiencing extreme exclusion and may not present with typical healthcare utilization patterns (11). In our recent scoping review of care transitions models for high-need patients (36), we found that measurement of continuity was either absent or lacking from prominent studies. In the first year of operation, our medical respite program achieved a measurement of continuity by showing a 15% improvement in primary care provider linkage between admission to the respite program and first post-hospitalization visit within 7 days. This concrete quantification of continuity, which is the primary outcome of a large randomized controlled trial of complex care coordination for people experiencing homelessness in Toronto, Canada (37), and its marked increase because of our intervention, was relatively easy to track within our model, and indicates a direct benefit of this CSC model of care for a high-need population.

Our partner feedback showed that although patients were admitted to the respite program with clear clinical need during the initial period of care transition, the greatest long-term risk patients faced was in relation to predominantly high acuity social needs. The extension of acute care hospital collaboration into the post-discharge space ensured that discharge failures were remedied promptly so that social sector providers could facilitate wraparound treatment alongside collaborative primary care treatment. By understanding and quantifying our patients' social risk, our program is also able to optimize data collection of social determinants that is required but challenging for our acute care hospital partners to aggregate, a new facet of US-based Federal regulatory requirements.

## Limitations

Our study is limited due to its small size, and nascent nature of our initial findings. Although our extraction of data from a social sector source is practical and offers an easily implemented framework for similar programs with limitations to health sector records, quality of data is limited. Although we possessed University at Buffalo Institutional Review Board approval to perform retrospective review of informal program data for the purpose of baseline measurement and quality improvement, hypothesis testing was not performed due to the small sample size. Additionally, our informal collection of observations on the implementation process could be bolstered by formal methods, such as through regular, formal focus groups and subsequent qualitative analysis.

## Conclusion

This example of a collaborative medical respite program formation illustrates the potential for CSC implementation and adds to the call for further development of implementation strategies that address external organizational influences to better understand the external domain integral to CSC and thus to better meet the demands of our complex patients and agencies where they receive care (38). Our evaluation has demonstrated that by developing shared vision and corresponding workflow, providers from cross-sector agencies may gain clarity about their roles, and in doing so, improve long-term effectiveness and sustainability of the program through normalization of collaborative tasks.

Future research will focus on CSC as an integral facet of the intervention, not simply a domain to consider during implementation, and utilize innovative frameworks which specifically address CSC interventions as an imminent need in current patient care, such as the Consolidated Framework for Collaboration Research (CFCR) (39), a developing implementation science framework focused on community engagement. Our collaborative model illustrates the importance of elements of CSC that bridge the vast spaces between sectors outlined in this emerging framework and similar body of literature, such as close attention to who is actively engaged (group composition), how a shared vision is implemented (structure and internal processes), and how to optimize relationships toward mutual empowerment (activities in community and collaboration). The intentional focus on community engagement of both CFCR and our model, instead of cross-agency competition for clients, could help strengthen further expansion of medical respite as an evidence-based model that requires CSC for successful implementation and leads to beneficial outcomes for our most vulnerable patients.

## Data Availability

The data analyzed in this study is subject to the following licenses/restrictions: Data was collected during program operations in a private institution and is not publicly available. Queries should be directed to Amanda Joy Anderson, ajanders@buffalo.edu.
